# MSH2/MSH6 Complex Promotes Error-Free Repair of AID-Induced dU:G Mispairs as well as Error-Prone Hypermutation of A:T Sites

**DOI:** 10.1371/journal.pone.0011182

**Published:** 2010-06-17

**Authors:** Sergio Roa, Ziqiang Li, Jonathan U. Peled, Chunfang Zhao, Winfried Edelmann, Matthew D. Scharff

**Affiliations:** Department of Cell Biology, Albert Einstein College of Medicine, New York, New York, United States of America; University of Minnesota, United States of America

## Abstract

Mismatch repair of AID-generated dU:G mispairs is critical for class switch recombination (CSR) and somatic hypermutation (SHM) in B cells. The generation of a previously unavailable *Msh2^−/−^Msh6^−/−^* mouse has for the first time allowed us to examine the impact of the complete loss of MutSα on lymphomagenesis, CSR and SHM. The onset of T cell lymphomas and the survival of *Msh2^−/−^Msh6^−/−^* and *Msh2^−/−^Msh6^−/−^Msh3^−/−^* mice are indistinguishable from *Msh2^−/−^* mice, suggesting that MSH2 plays the critical role in protecting T cells from malignant transformation, presumably because it is essential for the formation of stable MutSα heterodimers that maintain genomic stability. The similar defects on switching in *Msh2^−/−^*, *Msh2^−/−^Msh6^−/−^* and *Msh2^−/−^Msh6^−/−^Msh3^−/−^* mice confirm that MutSα but not MutSβ plays an important role in CSR. Analysis of SHM in *Msh2^−/−^Msh6^−/−^* mice not only confirmed the error-prone role of MutSα in the generation of strand biased mutations at A:T bases, but also revealed an error-free role of MutSα when repairing some of the dU:G mispairs generated by AID on both DNA strands. We propose a model for the role of MutSα at the immunoglobulin locus where the local balance of error-free and error-prone repair has an impact in the spectrum of mutations introduced during Phase 2 of SHM.

## Introduction

Immunoglobulin (Ig) genes undergo somatic hypermutation (SHM) to produce high affinity antigen binding sites and class switch recombination (CSR). These processes allow the antibodies to bind antigens strongly and to carry out different effector functions and be distributed throughout the body so that they can inactivate pathogens and other toxic substances. Both SHM and CSR are initiated by activation-induced cytidine deaminase (AID), which is highly expressed in germinal center B cells [1] and is primarily targeted to antibody variable (V) and switch (S) regions. When mistargeted, these processes lead to B cell malignancies [2]. According to a widely accepted model [3], [4], in Phase 1 of this process, AID preferentially deaminates the C residues in WRC (W = A or T, R = A or G) hotspot motifs in antibody genes and converts dC to dU in single stranded DNA. The uracils are then either replicated over to produce transition mutations or processed by short patch base excision repair (BER) to produce both transitions and transversions. Alternatively, in the Phase 2, the dU:G mismatches are recognized by the mismatch repair (MMR) complex that recruits low fidelity polymerases to resolve the AID-generated dU:G mispairs and generate additional mutations, especially at A:T residues, in the Ig V and S regions.

In view of their importance in maintaining genomic stability [5], [6], it is paradoxical that MMR and BER play important roles in generating the DNA mutations and double-strand breaks that are required for SHM and CSR. MSH2 dimerizes with MSH6 or MSH3 to form MutSα or MutSβ heterodimers, respectively, that play distinct, though partially overlapping, functions during mismatch repair [5]. Consistent with the central role of MSH2, genome instability has been shown to be greater in *Msh2^−/−^* mice than in the absence of either of its heterodimerization partners, MSH6 or MSH3 [7]. *Msh2^−/−^* mice die predominantly from T cell lymphomas but have some intestinal tumors [8] and several members of recently described kindreds carrying biallelic mutations in Msh2 have developed T cell lymphomas [9]. Interestingly, *Msh6^−/−^* mice exhibit a different tumor phenotype, dying predominantly from B cell lymphomas [10], despite the fact that MSH2 and MSH6 form a heterodimer to recognize single base mismatches and initiate MMR. The special relevance of MSH2/MSH6 in B cells is further evidenced by the findings that mice deficient in either MSH2 or MSH6 exhibit comparable decreases in CSR and losses of mutations at A:T bases [11]–[15]. Deficiency in MSH3, however, did not show any significant phenotype in CSR or SHM [12], [15], [16], strongly suggesting that the MSH2/MSH6 is the critical heterodimer that initiates MMR during SHM or CSR.

Unlike MSH2, the N-terminal regions of MSH6 and MSH3 have an additional conserved domain that binds PCNA [17]–[20], a sliding clamp that plays a role in many repair processes and in SHM and CSR [21], [22]. In addition, crystal structures of MutSα bound to DNA indicate that only MSH6 interacts directly with the mismatch [23] and other evidence suggests that MSH6 has scaffolding functions independent of its enzymatic activity that influence AID targeting during SHM [24]. The differences in the tumor phenotype of the MSH2 and MSH6 deficient mice, and the fact that they have some different functional domains, raises the possibility that disruption of both MSH2 and MSH6 would intensify genomic instability in B or T cells and further impair SHM and CSR. In fact, global genomic instability was moderately higher in *Msh2^−/−^Msh3^−/−^* and *Msh3^−/−^Msh6^−/−^* than in single *Msh2^−/−^* mice [7], which also suggests additional functions of MSH6 that are independent of MSH2. To further explore the roles of MSH2 and MSH6 in tumorigenesis, in CSR and in SHM at A:T bases, it would be useful to analyze mice simultaneously lacking both proteins and therefore completely deficient in MutSα complex dependent functions [7]. Additionally, since MSH2/MSH6 might compete with the uracil DNA-glycosylase (UNG) that initiates BER for access to the U in the dU:G mismatch [4], [25], complete deficiency of MutSα might lead to a significant increase in BER. However, the genes for *Msh2* and *Msh6* are very closely linked on chromosome 17 and mice that are homozygous deficient in both genes have not been available. In the present study, we generated such homozygous doubly deficient mice via extensive breeding and examined the impact of the loss of Mutα on lymphomagenesis, SHM and CSR.

## Results and Discussion

### Generation of *Msh2^−/−^Msh6^−/−^* and *Msh2^−/−^Msh6^−/−^Msh3^−/−^* deficient mice

**Figure 1:**
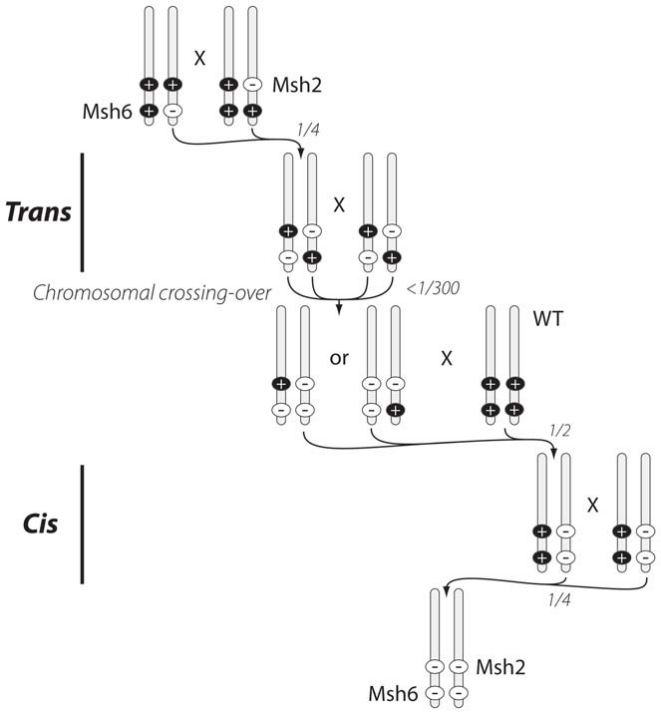
The generation of *Msh2^−/−^Msh6^−/−^* mice. *Msh2* and *Msh6* loci are separated by less than one megabase at murine chromosome 17. The “+” symbol represents the wild type allele and the “−” symbol represents the knockout allele. “Trans” indicates that the *Msh2* and the *Msh6* knockout alleles are in different chromosomes, and “Cis” indicates that the *Msh2* and the *Msh6* knockout alleles are in a same chromosome.

The *Msh2* and *Msh6* genes are very closely linked on murine chromosome 17 and are separated by a distance of less than one megabase. Therefore, it is extremely difficult to generate double knockout mice using the usual double heterozygous mating scheme [7]. To circumvent this problem, we first bred *Msh2^+/−^* mice with *Msh6^+/−^* mice that had been fully backcrossed to the C57BL/6 strain to generate *Msh2^+/−^Msh6^+/−^* mice in which *Msh2* mutant allele and *Msh6* mutant allele were in a *trans* position. We then inter-bred *trans Msh2^+/−^Msh6^+/−^* mice and screened for progeny that had undergone meiotic recombination between these two loci so that one locus was homozygous and the other locus was heterozygous (Figure 1). After screening more than 300 pups, one such *Msh2^−/−^Msh6^+/−^* mouse was identified. This *Msh2^−/−^Msh6^+/−^* mouse was bred with a wild type C57BL/6 mouse to generate *Msh2^+/−^Msh6^+/−^* mice in which the defective *Msh2^−^* and *Msh6^−^* genes were in *cis* (Figure 1). This strain was further backcrossed to C57BL/6 mice for three generations. Then, these *cis* double heterozygous mice were bred with each other to generate the *Msh2^−/−^Msh6^−/−^* doubly deficient mice at the expected frequency of 25% (Figure 1 and data not shown). Since the *Msh3* gene is on murine chromosome 13, triple heterozygous *Msh2^+/−^Msh6^+/−^Msh3^+/−^* were generated by breeding *cis Msh2^+/−^Msh6^+/−^* with *Msh3^+/−^* mice as a preliminary step to the generation of triple knockout animals.

### MSH2 is a limiting factor in the susceptibility to lymphomagenesis

**Figure 2:**
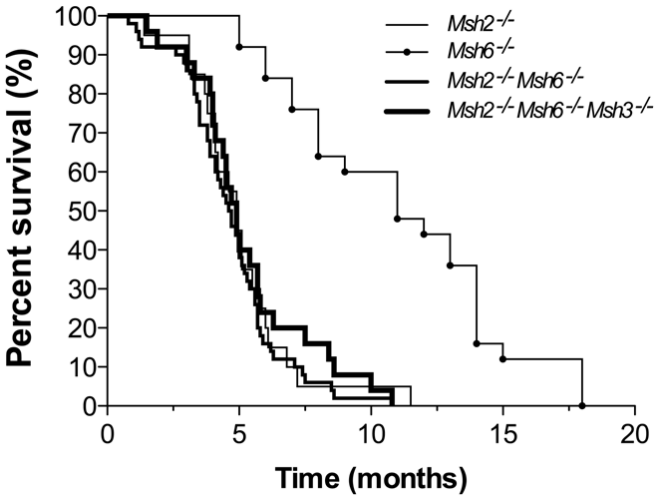
Comparison of the survival curves among MMR deficient mice. *Msh2^−/−^* (N = 20), *Msh2^−/−^Msh6^−/−^* (N = 50) and *Msh2^−/−^Msh6^−/−^Msh3^−/−^* (N = 25) mice die faster than *Msh6^−/−^* (N = 25) (log-rank test *p*<0.001). The survival curve of *Msh6^−/−^* mice was published previously [10].

As shown in Figure 2, *Msh2^−/−^Msh6^−/−^* mice had similar life span (median survival of 4.7 months) to *Msh2^−/−^* mice (median survival of 4.9 months) (log-rank test *p* = 0.5659). This early lethality was significantly different (log-rank test *p*<0.001) from the previously reported survival of *Msh6^−/−^* mice, which preferentially develop B cell lymphomas after a longer latency [10]. When *Msh2^−/−^Msh6^−/−^* mice became moribund, they were euthanized and necropsied. Large tumors were observed in all animals, and four out of four cases analyzed by immunohistochemistry were B220^–^CD3^+^ T cell lymphomas (see Figure S1). The fact that *Msh2^−/−^* [8] and *Msh2^−/−^Msh6^−/−^*, but not *Msh6^−/−^* mice [10], develop an indistinguishable T cell lymphoma phenotype suggests that T cells are especially susceptible to lymphomagenesis in the absence of normal MutSα (MSH2/MSH6) and that MutSβ (MSH2/MSH3) is sufficient to protect T cells from malignant transformation. Consistent with this idea, *Msh2^−/−^Msh6^−/−^Msh3^−/−^* mice had similar life span (median survival of 4.9 months) to *Msh2^−/−^* mice (log-rank test *p* = 0.8404). The differences in susceptibility to malignant transformation of *Msh2^−/−^* and *Msh6^−/−^* mice and the protective role of MSH2 in both T and B cells is further supported by the finding that in the absence of T cells, lack of MSH2 results in B cell lymphomas, which was otherwise obscured by the early appearance of T cell lymphomas [26]. This does not preclude individual and distinct functions for MSH2, and especially for MSH6 in B cells, but it does suggest that MSH2 plays the dominant role in protecting T and B cells from malignant transformation, presumably because it is shared by both MutSα and MutSβ. Since the deficiency of MSH3 is associated with very late onset of lymphomas that is indistinguishable from wildtype animals [27], MutSα appears to be much more protective than MutSβ suggesting that either the instability in microsatellites or the single base changes that are repaired by MutSα are more important in malignant transformation than larger mismatches or the microsatellites that are repaired by MutSβ.

### The MSH2/MSH6 heterodimer (MutSα) is required for efficient CSR

**Figure 3:**
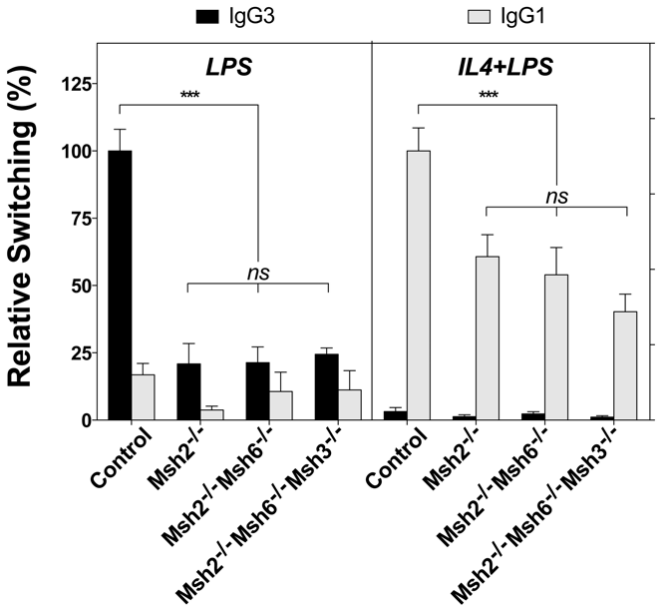
Class switch recombination analysis. *Msh2^−/−^* (N = 2), *Msh2^−/−^Msh6^−/−^* (N = 4) and *Msh2^−/−^Msh6^−/−^Msh3^−/−^* (N = 3) mice were assayed in five independent experiments and showed a similar decrease in the relative switching to IgG3 and to IgG1 compared to their control littermates (N = 11). The average switching of the wildtype mice in each experiment was defined as 100% and the data shown represent the mean ± SD. *P* values were calculated using two-tailed unpaired Student's *T* tests. ***p<0.001 was considered statistically significant; *ns*, no significant, *p>0.05*.

Numerous studies based on single mutant mice support the notion that MSH2/MSH6 is the major MutS complex involved in CSR and SHM. To test whether there are heterodimer-independent functions of MSH6 that may positively or negatively contribute to CSR, *Msh2^−/−^Msh6^−/−^* and *Msh2^−/−^Msh6^−/−^Msh3^−/−^* mice were used to assay CSR *ex vivo* and compared to single *Msh2^−/−^* mice. Primary splenic B cells from these mice were stimulated with LPS to induce switching to IgG3, or with LPS and IL4 to induce switching to IgG1. Figure S2 shows flow cytometry results from one representative experiment in which the double knockout mice had a reduced frequency in switching to both IgG3 and IgG1. The data from two *Msh2^−/−^*, four *Msh2^−/−^Msh6^−/−^*, three *Msh2^−/−^Msh6^−/−^Msh3^−/−^* mice, and their combined eleven littermate controls showed that the double and triple deficient B cells had ∼75% reduction in the frequency of switching to IgG3 (*p*<0.001) and ∼50% reduction in switching to IgG1 (*p*<0.001) compared to the control mice (Figure 3). This reduction in the rate of CSR was indistinguishable from the decrease detected here for MSH2 single deficient mice and comparable to what we, and others, have previously reported for *Msh2^−/−^* and for *Msh6^−/−^* mice [14]–[16], [28]. This result argues against the existence of MSH2-independent functions of MSH6 during CSR, and confirms that the efficiencies of switching are dramatically impaired when the MutSα complex cannot be stabilized as a heterodimer at AID-damaged DNA. Although there appears to be a trend towards a decrease from *Msh2^−/−^* to *Msh2^−/−^Msh6^−/−^Msh3^−/−^* mice, the differences are not significant and are consistent with the earlier finding that MutSβ and MSH3 do not have an important role in CSR [15], [16].

### MSH2/MSH6 heterodimer (MutSα) favors hypermutation at A:T sites

**Figure 4:**
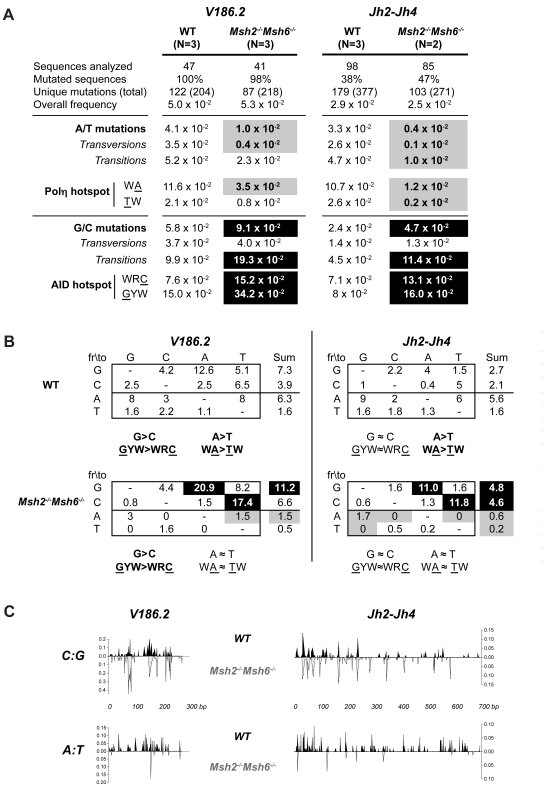
Effects of MSH2/MSH6 deficiency on somatic hypermutation of V186.2 and Jh2-Jh4 regions. (A) Global analysis of unique mutation frequencies corrected for base composition. All mutation frequencies were calculated according to the standardization method suggested in SHMTool [30]. N denotes the number of animals assayed in each group. Counts of unique mutations and relative frequencies of mutations are shown in Figure S3. Black boxes denote statistically significant increase (*p*<0.05) of mutation frequency compared to WT; gray boxes denote significant decrease (*p*<0.05). For V186.2 region, the RT + PCR error rate was estimated to be 0.14×10^−2^ mutations/base because one G-to-A mutation was detected in the Cγ1 segment (39 bp) adjacent to the V186.2 region. (B) The spectrum of base substitutions presented as frequencies of mutation (x10^−2^ mutations/base) and corrected for base composition. The site in hotspot motifs that was scored for mutation is underlined. W  =  A/T, R  =  A/G and Y  =  C/T. (C) Distribution of mutations in the V186.2 (273 bp) and Jh2-Jh4 (693 bp) regions. The x-axis indicates the mutation positioning and the y-axis indicates the frequency of sequences mutated at each site. Mutation data from wildtype or *Msh2^−/−^Msh6^−/−^* mice is presented above or below the x-axis, respectively.

Similarly to CSR, a requirement for both MSH2 and MSH6 to resolve AID-generated mismatches was observed when we analyzed SHM in *Msh2^−/−^Msh6^−/−^* mice. In young NP-immunized mice, unique mutations were compiled from splenic B cells that had mutated their V186.2 gene, which is a member of the J558 immunoglobulin gene family that dominates the response to NP-immunization [29]. In old unimmunized mice, unique mutations were compiled from the Jh2-Jh4 region of B220^+^PNA^high^ Peyer's patch B cells, which is an intronic region that accumulates a large number of mutations but presumably is not subject to selective pressure during the immune response. To calculate mutation frequencies, the accumulated number of unique mutations were divided by the theoretical maximum number of the corresponding type of mutation so as to correct for base composition [30]. As shown in Figure 4A, simultaneous deficiency of MSH2 and MSH6 produced similar effects in the SHM of both V186.2 and Jh2-Jh4 regions. In contrast to previously studied single deficient Msh2 [11], [31], [32] or Msh6 models [16], [24], which exhibited reduced overall frequencies of mutation, there was not a decrease in the overall frequency of unique mutations in the *Msh2^−/−^Msh6^−/−^* B cells studied here (Figure 4A). Since MutSα complexes preserve genomic stability in chronically stimulated B cells [33] and a survival disadvantage might affect the overall accumulation of mutations [34], [35], we can not rule out the possibility that the combined deficiency of both Msh2 and Msh6 made chronically stimulated B cells less efficient in activating apoptotic checkpoint programs allowing them to survive longer and accumulate as many mutations as wildtype cells. However, the even more dramatic scenarios lacking both MMR and BER (i.e. *Msh2^−/−^Ung^−/−^* [34] and *Msh6^−/−^Ung^−/−^* [35]) also exhibited normal overall mutation frequencies, suggesting that the mechanisms downstream of AID do not introduce additional mutations but rather modulate the initial spectrum. In *Msh2^−/−^Msh6^−/−^* B cells the spectrum of mutations was dramatically changed so that the balance in the frequencies of mutations at A:T versus C:G sites was shifted in different directions (Figure 4A), suggesting opposing effects of MMR in the resolution of each type of mutation during the Phase 2 of SHM.

Transitions and transversions at A:T pairs were significantly reduced in *Msh2^−/−^Msh6^−/−^* mice compared to their wildtype littermates (Figure 4A and B). Since the recruitment of Polη, which preferentially targets WA/TW motifs [36]–[38], is crucial during Phase 2 of hypermutation [21], [22], [32] and MSH2/MSH6 can mediate its recruitment and stimulation [39], we searched for hallmarks of impaired Polη mutation. Analysis of the spectrum of mutations showed that targeting of A residues within WA motifs was significantly impaired in *Msh2^−/−^Msh6^−/−^* mice (Figure 4). There was a less robust, though still significant, decrease in mutations at T residues and TW motifs in the Jh2-Jh4 region, but this was only marginally significant in the V186.2 region, most likely due to the low number of mutations at T sites. The preferential decrease in A:T mutations within WA/TW motifs is consistent with previous data from *Msh2^−/−^* [11], [31], [32], [40], *Msh6^−/−^* [16], [24] and *Msh3^−/−^Msh6^−/−^* [12] mice and strongly suggests that MutSα is the major contributor of A:T mutations at the Ig locus. The presence of residual A:T mutations in the sequences from *Msh2^−/−^Msh6^−/−^* B cells support previous evidence of an alternative pathway, presumably long-patch BER since these mutations in A:T are not seen in *Msh2^−/−^Ung^−/−^* and *Msh6^−/−^Ung^−/−^* mice [34], [35].

### The MSH2/MSH6 heterodimer (MutSα) has an error-free repair impact in hypermutation at C:G sites

**Figure 5:**
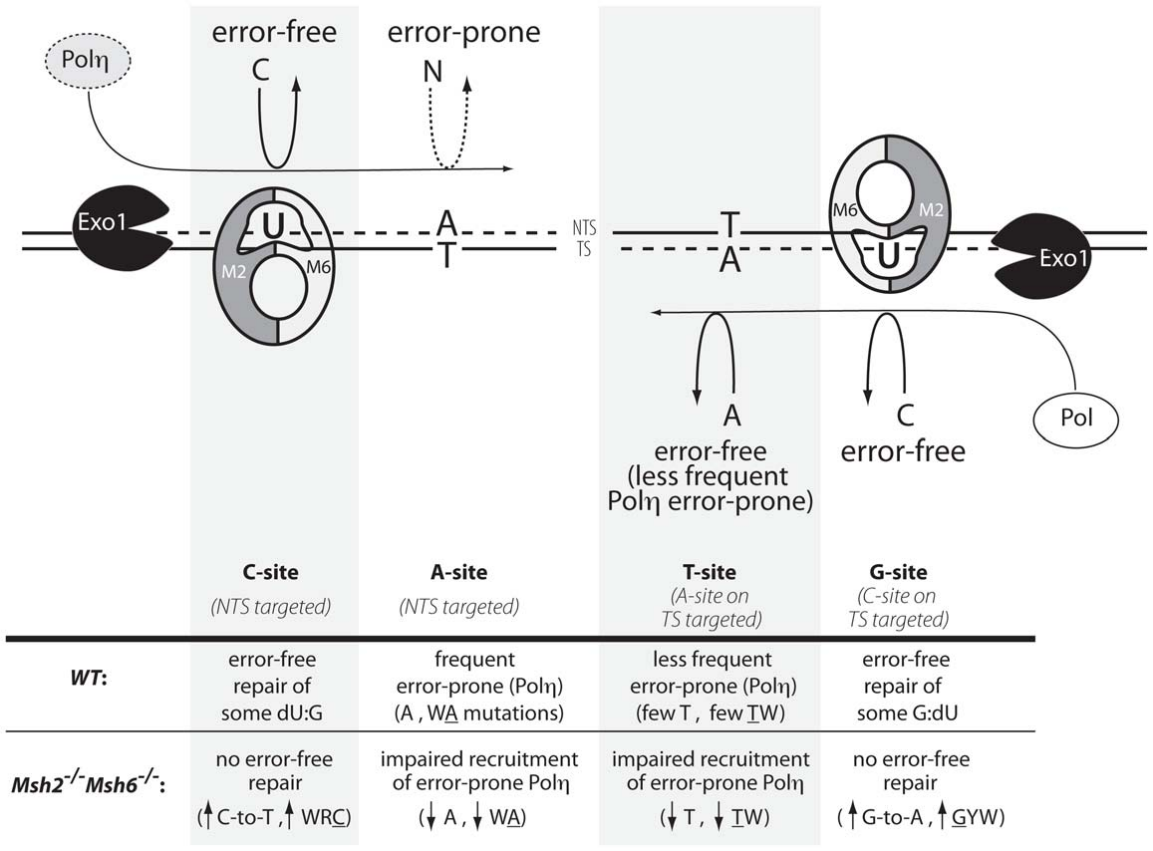
Model for MMR resolution of AID-generated dU:G mispairs. In the DNA deamination model, MSH2/MSH6 recognizes the initial AID-generated dU:G lesion, the damaged strand is excised by Exo1, and Polη is preferentially recruited to the nontranscribed strand (NTS) to generate mutations at A sites within WA motifs. Fewer T sites within TW motifs are mutated, contributing to the singular A≫T and WA≫TW biases that characterize SHM and reflect the reduced targeting of A sites by Polη in the transcribed strand (TS). We highlight here the error-free repair effect of MMR at the dU:G mispairs within the excised patch, which is revealed in *Msh2^−/−^Msh6^−/−^* mice by a significant increase in unrepaired dU:G lesions that are replicated as transitions at C:G sites in WRC/GYW hotspots (i.e. C-to-T within WRC in the NTS, and G-to-A within GYW as a reflection of C-to-T mutations in the TS).

A relative increase in C:G mutations, primarily in transitions, has been described in MMR deficient mice [11]–[13], [16], [24], [31], [32], [40], [41], but it is not clear if this can be explained solely by the dramatic reduction of mutations at A:T sites or if there is also an absolute increase in the frequency of C:G mutations. This question was recently examined in B cells from *Msh2^−/−^* mice, where a relative, but not an absolute, increase of transitions at C:G sites was reported [40]. In *Msh2^−/−^Msh6^−/−^* mice, however, we found both a relative and an absolute increase in the frequency of transition mutations at C:G pairs within WRC/GYW hotspots (Figure 4A and S3). One possible explanation for the discrepancy between the absolute frequencies of C:G mutations from the single and the double knockout may lay, as discussed before, in the overall increase in C:G mutations observed in *Msh2^−/−^Msh6^−/−^* mice compared to *Msh2^−/−^* mice [40]. This increase in C:G transitions can most readily be explained by replication of dU:G lesions that were no longer detected and processed by the missing MMR proteins to introduce C-to-T or G-to-A mutations (Figure 5). This suggests that in wildtype mice MMR carries out error-free repair of some of the uracils by copying with fidelity the opposing G during the mutagenic patch-repair mechanism that mobilizes Polη to cause error-prone mutations in A:T sites (Figure 5). Such a possibility is in fact consistent with the much less error-prone activity of Polη when copying C:G pairs compared to its clear error-prone tendency when using T as template [42].

As a consequence of the accumulation of unrepaired AID-generated mutations at WRC/GYW hotspots and the loss of A:T mutations (Figure 4A and 4B), both V186.2 and Jh2-Jh4 sequences from *Msh2^−/−^Msh6^−/−^* mice exhibited a more restricted pattern in the distribution of mutations compared to wiltdtype (Figure 4C). Similar results in single *Msh2^−/−^* [11], [32] and *Msh6^−/−^* mice [16], [24] support our interpretation that in the absence of MMR some of the entry points for AID activity remain as mismatches until replication.

On the other hand, the absolute frequency of transversions at C:G sites was not affected in the absence of MSH2/MSH6 (Figure 4A). Since BER is a major contributor of transversions at C:G pairs [43], this unaltered resolution of certain dU:G mismatches into transversions is consistent with continued error-prone BER activity that is independent of MMR and is not enhanced in the absence of MSH2/MSH6. This result suggests that there is little competition between these two repair processes as they act on the Ig locus to deal with AID induced mutations and supports previous evidence for a noncompetitive model [40]. Our data does not rule out the existence of a cooperation between MSH2 and UNG in generating some C:G transversions [40], [44], but does suggest that the impact of such synergistic mechanism can be masked by the independent activity of error-prone BER in the absence of both MSH2 and MSH6.

### Strand bias signatures in *Msh2^−/−^Msh6^−/−^* mice

Polη is known to be responsible for the A≫T strand bias signature of SHM (mutations of A exceeding mutations of T) [36]–[38], which results from the preferential targeting of A nucleotides for mutation within WA motifs on the non-transcribed strand (Figure 5). Here, we found that both V186.2 and Jh2-Jh4 regions from wildtype mice exhibited the expected significant A≫T (*p*<0.001) and WA≫TW bias (*p*<0.001) (Figure 4A and B). However, the dramatic decrease of A:T mutations in *Msh2^−/−^Msh6^−/−^* B cells made this strand bias undetectable (A≈T and WA≈TW, *p*≥0.1708) (Figure 4B), suggesting that Phase 2 mutations were introduced by a preferential activity of error-prone MMR on the nontranscribed strand (Figure 5).

As described above, transitions at C within WRC sites were increased in *Msh2^−/−^Msh6^−/−^* mice (Figure 4), which could be attributable to unrepaired mutations in the nontranscribed strand. This increase was comparable to the increase in transitions at G within the complementary GYW motif, which reflects the targeting of WRC in the transcribed strand. This observation supports the notion that error-free MMR of dU:G mispairs occurs in both strands, as opposed to the previously discussed MMR error-prone activity that preferentially targets A sites in the nontranscribed strand (Figure 5).

Independently of the observation that MMR affected the processing of dU:G mispairs on both strands, in both WT and double-deficient mice the V186.2 region exhibited an intrinsic strand bias of mutations at WRC on the transcribed strand (compiled as GYW mutations) exceeding mutations at WRC on the nontranscribed strand (Figure 4, GYW>WRC, *p*<0.05). The intronic Jh2-Jh4 region, however, did not show this bias in C:G or hotspot mutations. Similarly, the existence of a subtle G>C bias during SHM has been recently revealed in antigen-selected V(D)Js but not in non-antigen selected JH4 introns [38]. Since recent *in vivo* evidence suggests that AID shows an initial preference for C sites on the nontranscribed strand (initial C>G bias) [34], [35], [45], it still remains unclear how the repair process is able to override this initial bias and prompt a scenario with no bias (C≈G) or a subtle G>C bias. According to the mutation spectrum described here for *Msh2^−/−^Msh6^−/−^* mice, it seems unlikely that MMR is responsible for this because the patterns of C≈G in the Jh2-Jh4 region or G>C in the V186.2 region were preserved even in the absence of MutSα. The interpretation of these findings could be further complicated if the interplay between MMR and BER to repair AID induced mutations differs between G1/G2 phases of the cell cycle, when there is no postreplicative repair, and S, when uracils may be introduced genome wide as a result of normal replication [3], [4], [25], [44], [46].

The idea that mismatches at G or C in the immunoglobulin locus may undergo error-free repair [13], [32], [47] and the existence of a balance between error-prone and error-free repair have been raised before [48]. Here, we present evidence suggesting that a significant number of AID-generated uracils in both strands of the immunoglobulin locus can be repaired with high fidelity by MutSα complexes. This error-free repair appears to be locally balanced with a strand biased error-prone introduction of mutations at A:T sites, which is largely impaired in the absence of MutSα complexes. CSR is also severely affected in mice lacking MutSα complexes, although a number of switching events remain, presumably due to the unique action of BER. In summary, the overall impairment of the immune response and the increased susceptibility to lymphomagenesis that is associated with the absence of MSH2/MSH6 heterodimers highlight the critical role of MutSα in the generation of antibody diversity in B cells and the protection from malignant transformation of both B and T cells.

## Materials and Methods

### Mice

The single *Msh2^−/−^*, *Msh6^−/−^* and *Msh3^−/−^* mouse lines were generated and reported previously [10], [27], [49]. These mice were fully backcrossed to C57BL/6 (The Jackson Laboratory) in a barrier facility and used in this study to generate the double *Msh2^−/−^Msh6^−/−^* and triple *Msh2^−/−^Msh6^−/−^Msh3^−/−^* mice. All mouse experiments were approved by the Albert Einstein College of Medicine Animal Use Committee (Protocol number 20080801).

### Survival Curves

The life spans of *Msh2^−/−^Msh6^−/−^* and *Msh2^−/−^Msh6^−/−^Msh3^−/−^* mice were compared with those of contemporary *Msh2^−/−^* mice and previously published *Msh6^−/−^* mice [10]. Log-rank (Mantel-Cox) test from GraphPad Prism 5.0a was used to perform multiple comparisons of the survival curves and their overall trend.

### *Ex vivo* Class-Switching Assay

Splenic B cells were obtained from 6- to 10-week-old mice after complement-mediated T cell depletion [28] and 0.5×10^6^ cells/ml were stimulated with 50 µg/ml LPS (Sigma) or 50 µg/ml LPS and 50 ng/ml rIL-4 (R&D) for four days. Surface IgM and IgG were analyzed with a FACSCalibur (Becton-Dickinson) and FlowJo software (Treestar).

### Hypermutation Analysis

To assay SHM in the V186.2 region, 2-month-old mice were immunized intraperitoneally with (4-hydroxy-3-nitrophenyl)acetyl (NP)30-CGG (chicken gamma globulin) (BioSearch Technologies) in alum (Pierce) and boosted 4 weeks after primary immunization. One week after the boost, RNA was extracted from splenic B cells with TRIzol (Invitrogen) and cDNA was synthesized using oligo(dT) and AccuScript high-fidelity reverse transcriptase (Stratagene). V186.2 region rearranged to the IgG1 constant region was amplified by nested PCR, cloned and sequenced as previously described [22]. To assay SHM in the Jh2-Jh4 region, 6-month-old un-immunized healthy mice were sacrificed and Peyer's patches were collected. DNA was extracted from sorted B220^+^PNA^high^ germinal center B cells and the Jh2-Jh4 intron region was amplified, cloned, and sequenced as previously described [24]. Alignment and analysis of unique mutations was done using SeqMan 5.07 (DNASTAR Inc.) and SHMTool (http://scb.aecom.yu.edu/shmtool) [30]. As consensus sequences, GenBank J00530.1 (nucleotides 224-496) for V186.2, NT_166318.1 (nucleotides 25521839-25521801) for Cγ1, and NT_166318.1 (nucleotides 25620784-25620092) for Jh2Jh4 were used. The presence of a significant A≫T and WA≫TW bias in the wildtype mutation data was used for quality assessment and largely ruled out the presence of contaminating PCR hybrids and artifactual mutation spectra [38].

## Supporting Information

Figure S1 Immunohistochemistry analysis of T cell lymphomas in *Msh2^−/−^Msh6^−/−^* mice. Displayed are representative images of a lymphoma from a single animal. Tumors from four animals yielded similar results. Mice that became moribund were euthanized and tissues were fixed in formalin, embedded in paraffin, and sectioned by the Histopathology Core Facility at Albert Einstein College of Medicine. (A) Hematoxylin and Eosin stain. For immunohistochemistry, after dewaxing, sodium-citrate antigen retrieval, and blockage of endogenous peroxidase and avidin/biotin activity, tissues were incubated with (B) Ready-To-Use polyclonal rabbit anti-CD3 (Dako) or (C) rat anti-B220 clone RA3-6B2 (BD Biosciences) at a 1∶20 dilution. Appropriate biotinylated secondary reagents were combined with DAB chromogen (Vector) and a light hematoxylin counterstain.
(4.07 MB TIF)

Figure S2 Representative *ex vivo* CSR experiment from splenic B cells stimulated with LPS and LPS plus IL-4. Representative FACS profiles in which splenic B cells from control, *Msh2^−/−^*, *Msh2^−/−^Msh6^−/−^* and *Msh2^−/−^Msh6^−/−^Msh3^−/−^* mice were cultured in the presence of LPS or IL4+LPS for 4 days prior to staining for surface IgG3, IgG1 and IgM. Live cells were gated based on FSC and SSC scatters, and the percentage of IgG^+^ B cells is indicated in each plot. Panels in columns show FACS data from four representative animals, one for each group, assayed in two comparable experiments.
(1.60 MB TIF)

Figure S3 Detailed mutations in the V186.2 and Jh2-Jh4 regions from WT and *Msh2^−/−^Msh6^−/−^* mice. (Left panel) Absolute number of unique mutations classified by base pair (fr/to indicates from y-axis to x-axis). (Middle panel) Percentages of the total number of unique mutations. A limitation to this normalization is that relative frequencies can be misleading and obscure changes in absolute frequencies [30]. (Right panel) We considered mutation frequency corrected for base composition and standardized by SHMTool to be a more useful summary statistic [30].
(1.40 MB TIF)
