# Supplementary figures and images for: MSH2/MSH6 Complex Promotes Error-Free Repair of AID-Induced dU:G Mispairs as well as Error-Prone Hypermutation of A:T Sites
Source: PLoS One. 2010 Jun 17;5(6):e11182. doi: 10.1371/journal.pone.0011182 (PMC2887398; doi:10.1371/journal.pone.0011182)

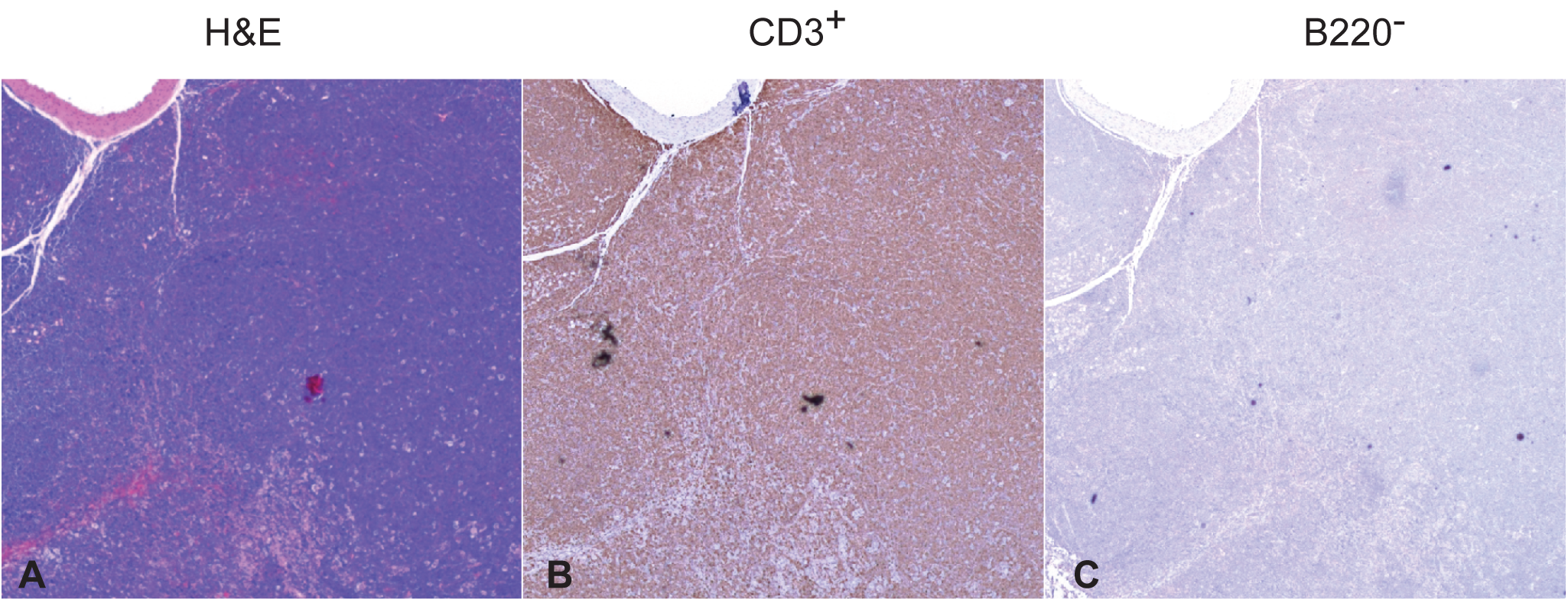

Supplement: Figure S1 — Immunohistochemistry analysis of T cell lymphomas in Msh2−/−Msh6−/− mice. Displayed are representative images of a lymphoma from a single animal. Tumors from four animals yielded similar results. Mice that became moribund were euthanized and tissues were fixed in formalin, embedded in paraffin, and sectioned by the Histopathology Core Facility at Albert Einstein College of Medicine. (A) Hematoxylin and Eosin stain. For immunohistochemistry, after dewaxing, sodium-citrate antigen retrieval, and blockage of endogenous peroxidase and avidin/biotin activity, tissues were incubated with (B) Ready-To-Use polyclonal rabbit anti-CD3 (Dako) or (C) rat anti-B220 clone RA3-6B2 (BD Biosciences) at a 1∶20 dilution. Appropriate biotinylated secondary reagents were combined with DAB chromogen (Vector) and a light hematoxylin counterstain. (4.07 MB TIF) [file pone.0011182.s001.tif]

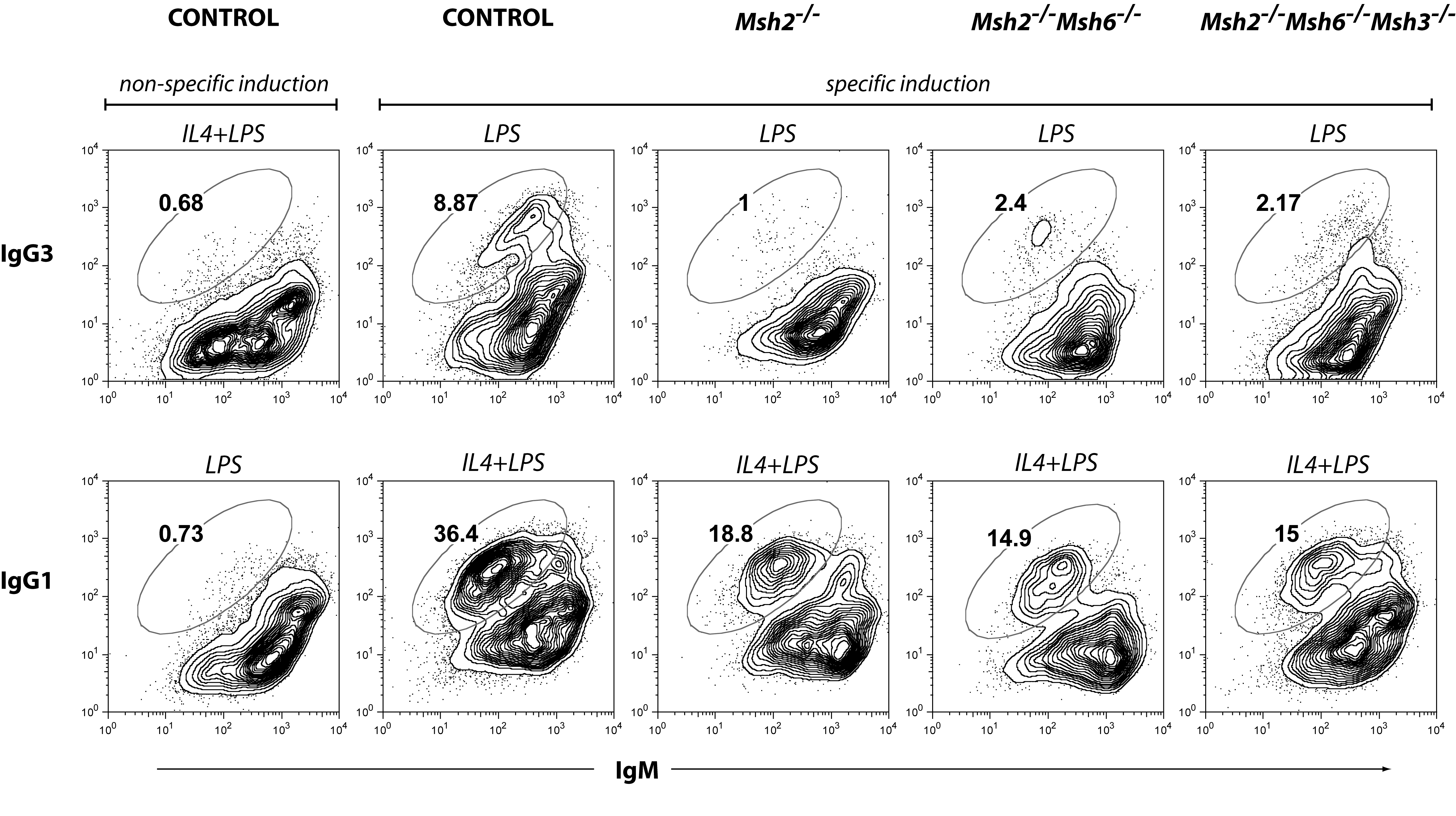

Supplement: Figure S2 — Representative ex vivo CSR experiment from splenic B cells stimulated with LPS and LPS plus IL-4. Representative FACS profiles in which splenic B cells from control, Msh2−/−, Msh2−/−Msh6−/− and Msh2−/−Msh6−/−Msh3−/− mice were cultured in the presence of LPS or IL4+LPS for 4 days prior to staining for surface IgG3, IgG1 and IgM. Live cells were gated based on FSC and SSC scatters, and the percentage of IgG+ B cells is indicated in each plot. Panels in columns show FACS data from four representative animals, one for each group, assayed in two comparable experiments. (1.60 MB TIF) [file pone.0011182.s002.tif]

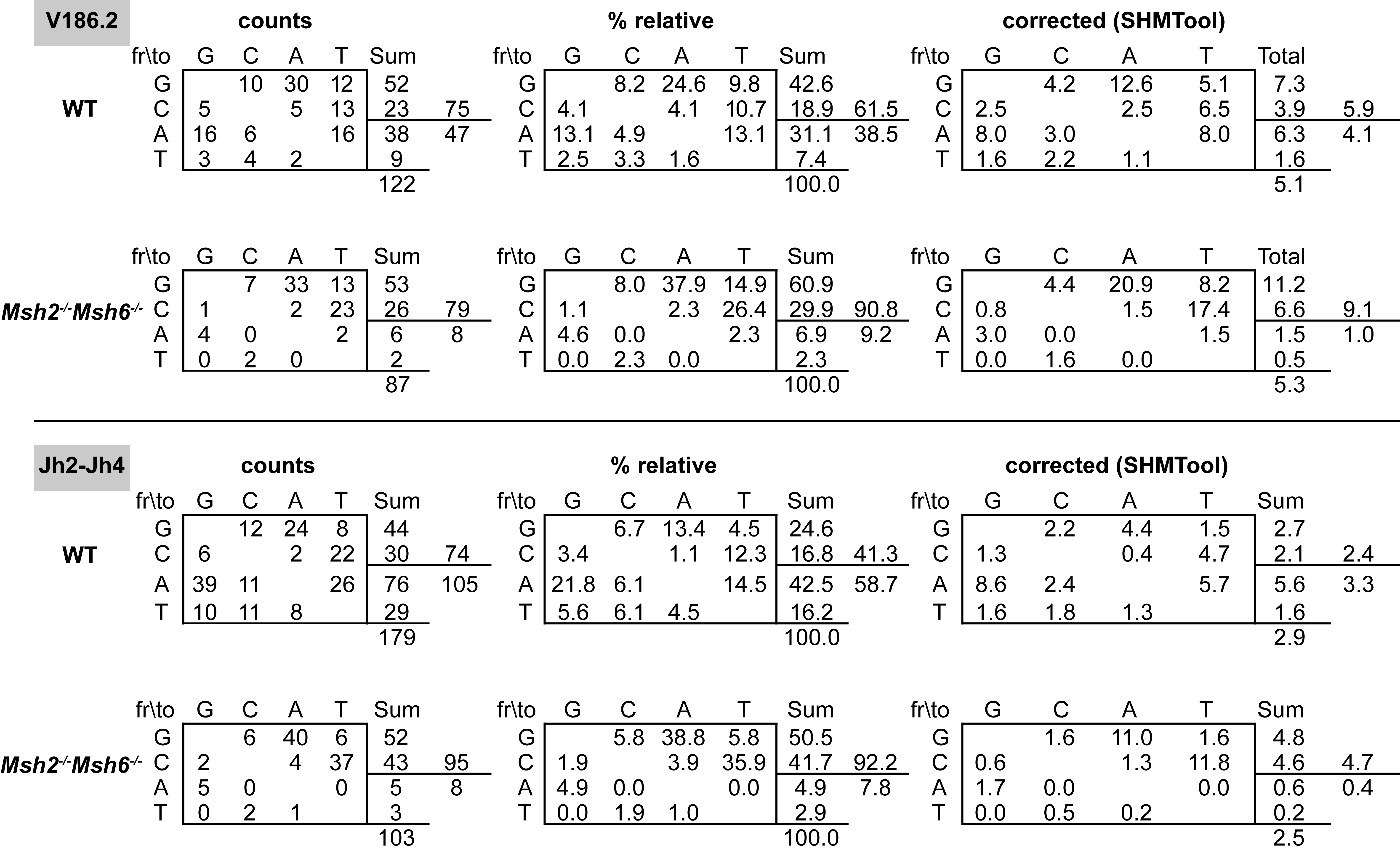

Supplement: Figure S3 — Detailed mutations in the V186.2 and Jh2-Jh4 regions from WT and Msh2−/−Msh6−/− mice. (Left panel) Absolute number of unique mutations classified by base pair (fr/to indicates from y-axis to x-axis). (Middle panel) Percentages of the total number of unique mutations. A limitation to this normalization is that relative frequencies can be misleading and obscure changes in absolute frequencies [30]. (Right panel) We considered mutation frequency corrected for base composition and standardized by SHMTool to be a more useful summary statistic [30]. (1.40 MB TIF) [file pone.0011182.s003.tif]
